# Editorial: Sexual function and dysfunction in men and women with diabetes

**DOI:** 10.3389/fendo.2025.1629375

**Published:** 2025-06-03

**Authors:** Adriana Coppola, Katherine Esposito, Carmine Gazzaruso

**Affiliations:** ^1^ Endocrinology Unit, Clinical Institute Beato Matteo, Vigevano, Italy; ^2^ Department of Advanced Medical and Surgical Sciences University of Campania Luigi Vanvitelli, Naples, Italy; ^3^ Department of Biomedical Sciences for Health, University of Milan, Milan, Italy

**Keywords:** sexual dysfunction, erectile dysfunction, diabetes, female sexual dysfunction (FSD), cardiovascular risk

Sexual dysfunctions are frequent in men and women with diabetes (1). Erectile dysfunction (ED) is a common and debilitating complication of diabetes mellitus (DM) affecting up to 75% of men with long-standing disease (2, 3). The pathophysiology of ED in diabetes is multifactorial, involving endothelial dysfunction, neuropathy, hormonal imbalances, and psychological stressors (2, 3). Recent evidence has broadened the understanding of the intricate mechanisms behind ED, as well as its clinical implications and potential management strategies (2, 3). Glycaemic control, insulin resistance, hypogonadism, obesity, oxidative stress, adipokine imbalance, and inflammation, often present in diabetic patients, synergistically contribute to endothelial damage and compromised penile vascular flow (1, 2, 4). The same risk factors responsible for ED lead to early atherothrombosis (4). In fact, one of the most clinically significant associations highlighted in recent years is the link between ED and cardiovascular disease (4). Several studies suggest that ED may be an early marker of systemic atherosclerosis and future cardiovascular events in diabetic men (4). Although some studies suggested a correlation between ED and angiographic extent of coronary artery disease in men with diabetes, other ones did not confirm this finding (5). Similarly, ED is independently associated with silent myocardial ischemia in asymptomatic patients, suggesting a role for ED as a sentinel symptom to search for occult cardiovascular disease (6). Therapeutic approaches have expanded beyond pharmacotherapy. Lifestyle interventions, particularly those aimed at weight reduction, physical activity, and dietary improvement, have shown promising effects (7). Maiorino et al. reported results from the MÈDITA trial, showing that adherence to a Mediterranean diet improved erectile function in newly diagnosed diabetic patients, independently of weight loss and glycaemic control (8). These findings emphasize the potential of non-pharmacologic interventions in reversing or mitigating ED, particularly when implemented early (7). Interventions such as smoking cessation, exercise, and weight management have a consistent, albeit modest, positive impact on erectile function (7). These effects are particularly relevant in the diabetic population, where the burden of comorbidities and vascular disease is high. A few information is available in the literature about the impact that diabetes treatment can have of the occurrence of ED. Finally, ED in diabetes may also reflect the presence of microvascular complications. A recent study investigated the association of ED with presence and outcomes of diabetic foot, finding that men with diabetic foot have a very high prevalence of ED, and that patients with both conditions have worse outcomes, including higher rates of foot ulcer recurrence and lower limb amputation (9). Several mechanisms, including hormonal, psychological, nervous, and vascular changes, can lead to female sexual dysfunction (FSD) in women with diabetes, even if these mechanisms are now little understood (1, 10). In addition, it is not known the real prevalence and incidence of female sexual dysfunction in diabetes, and whether there is an association between sexual dysfunction and cardiovascular diseases (10). This suggests that new studies should clarify many aspects of FSD in women with diabetes

This Research Topic presents five pivotal studies that can contribute significantly to our understanding of ED and FSD in diabetes. All these studies explore the pathophysiological mechanisms, genetic associations, risk factors, and potential treatment strategies for ED, offering valuable insights for both research and clinical practice. One of these studies gives some interesting information about sexual health in diabetic women. The review by Ma et al. provides a comprehensive updated analysis of the diverse pathophysiological processes underlying ED in diabetes. In addition, it discusses current and emerging treatment modalities, advocating for a multidisciplinary approach to management (Ma et al.). In the study by Feng et al. the authors employed a two-sample Mendelian randomization approach to investigate the causal relationships between commonly used antidiabetic medications and the risk of ED. The systematic review and meta-analysis by Dilixiati et al. synthesizes data from multiple studies to identify key risk factors associated with ED in diabetic men. This updated analysis reveals that factors such as age, smoking, duration of diabetes, poor glycaemic control, depression, metabolic syndrome, hypertension, some medications, and the presence of diabetic complications significantly increase the risk of ED. These findings underscore the importance of comprehensive risk assessment and management in diabetic patients to reduce the risk for ED (Dilixiati et al.). The cross-sectional study by Yu et al. explores the relationship between obesity, lipid profiles, and testosterone levels in a large study population. The study finds that indices such as the Visceral Adiposity Index, Chinese Visceral Adiposity Index, Triglyceride Glucose Index, and Lipid Accumulation Product are significantly associated with hypogonadism in type 2 diabetic men. Sensitivity and specificity of each of four indices were better or not worse than those of body mass index, homeostasis model assessment of insulin resistance and waist circumference. All four indices were effective predictors of hypogonadism (Yu et al.). These findings confirm that metabolic health plays a crucial role in hormonal balance and sexual function. In the study by Jiang et al. researchers examine the causal effects of type 2 diabetes, fasting insulin, and HbA1c on testosterone levels in both men and women with diabetes. The study concludes that metabolic parameters are causally associated with abnormal testosterone levels in both sexes (Yu et al.). The associations were independent of obesity, BMI, TG, LDL and Adiponectin (Jiang et al.). This research highlights the importance of glycaemic management in preserving hormonal health and sexual function in men and women with diabetes.

The studies presented in this Research Topic collectively advance our understanding of the complex interplay between diabetes and sexual dysfunction in men and women. They emphasize the multifactorial nature of ED, the significance of personalized medicine, and the need for comprehensive management strategies that address metabolic, hormonal, and psychological aspects. Future research should continue to explore these dimensions to develop effective interventions that improve the quality of life for diabetic patients experiencing sexual dysfunction in both sexes.
